# Supplementary material for: The genome sequence of the Map, Araschnia levana (Linnaeus, 1758) (Lepidoptera: Nymphalidae)
Source: Wellcome Open Res. 2025 Jul 31;10:398. [Version 1] doi: 10.12688/wellcomeopenres.24633.1 (PMC12402765; doi:10.12688/wellcomeopenres.24633.1)
Supplement: Peer review history

# Reviewer report: Reviewer response for version 1

**DOI:** 10.21956/wellcomeopenres.27147.r129547

**Published:** 2025-09-01

**Authors:** Rachel A Steward

**Affiliations:** 1 Lund University, Lund, Sweden

The authors present the genome of the Map butterfly, Araschnia levana. As the authors eloquently explain, this genome will be a valuable resource for the study of seasonal polyphenism and voltinism, especially in the context of climate-driven range expansions. 

 The genome, sequenced from a female sample from Bern, Switzerland, is highly complete, comprising 30 autosomes and both Z and W sex chromosomes, with nearly 99% complete Lepidopteran single copy orthologs. Similar to other Nymphalinae butterfly genomes published by Wellcome Open Research, painting with conserved Lepidopteran linkage groups suggest that the map genome has experienced very few chromosomal rearrangements (excluding ancient M17 M21 fusion). 

 The Genome Note presents a comprehensive overview of the sequenced genome, but does not present any annotation of gene features. Rather, the authors note that the genome will be annotated through Ensembl at EBI. 

Are sufficient details of methods and materials provided to allow replication by others?

Yes

Is the rationale for creating the dataset(s) clearly described?

Yes

Are the datasets clearly presented in a useable and accessible format?

Yes

Are the protocols appropriate and is the work technically sound?

Yes

Reviewer Expertise:

gene expression, alternative splicing, plant insect interactions, host use plasticity, seasonal polyphenisms

I confirm that I have read this submission and believe that I have an appropriate level of expertise to confirm that it is of an acceptable scientific standard.

# Reviewer report: Reviewer response for version 1

**DOI:** 10.21956/wellcomeopenres.27147.r129546

**Published:** 2025-09-01

**Authors:** Martijn Derks

**Affiliations:** 1 Wageningen University & Research, Wageningen, Gelderland, The Netherlands

This study presents a high-quality, chromosome-level genome assembly for the map butterfly *Araschnia levana*. The final haplotype 1 assembly spans 362.54 Mb with 99.09% placed into 32 chromosomal pseudomolecules, including the W and Z sex chromosomes. Haplotype 2 was assembled to scaffold level, and the mitochondrial genome was also reconstructed. The assembly has high completeness (98.8% BUSCO) and accuracy (QV ~62) with low levels of duplication. This reference genome will be valuable for studying seasonal polyphenism, wing patterning, and evolutionary processes in butterflies.

 One thing the authors could potentially look into is also if there are large structural variants between the haplotypes?

Are sufficient details of methods and materials provided to allow replication by others?

Yes

Is the rationale for creating the dataset(s) clearly described?

Yes

Are the datasets clearly presented in a useable and accessible format?

Yes

Are the protocols appropriate and is the work technically sound?

Yes

Reviewer Expertise:

Genomics, Bioinformatics

I confirm that I have read this submission and believe that I have an appropriate level of expertise to confirm that it is of an acceptable scientific standard.

# Reviewer report: Reviewer response for version 1

**DOI:** 10.21956/wellcomeopenres.27147.r128418

**Published:** 2025-08-30

**Authors:** Gulab D Khedkar

**Affiliations:** 1 Dr. Babasaheb Ambedkar Marathwada University, Aurangabad, Maharashtra, India

Review report on "The genome sequence of the Map, *Araschnia levana* (Linnaeus, 1758) (Lepidoptera: Nymphalidae)"

 This is a thoughtful and valuable genome-sequencing work that provides a high-quality, chromosome-level reference assembly for the Map butterfly, *Araschnia levana*. The authors have carefully executed research, resulting in an important resource that will be highly useful for the scientific community, particularly for researchers studying seasonal polyphenism and chromosomal evolution.

 Here is a review of the preprint, highlighting its strengths and offering some suggestions for improvement.

**Review Outcome**

**Strengths**

1. High-quality assembly: The assembly, particularly Haplotype 1, is of excellent quality. The use of long-read technology for initial assembly and Hi-C dat **a** for scaffolding to the chromosome level is a reliable approach. The assembly statistics, including a high scaffold N50 (12.61 Mb) and a high percentage of the assembly assigned to chromosomes (99.09%), demonstrate a near-complete representation of the genome.
2. Comprehensive methods: The methods section is thorough and transparent. They cite the protocols used, which may aid in reproducibility. The use of a female specimen allowed for the identification of both W and Z sex chromosomes, which is an important contribution.
3. Data quality assessment:  The assembly meets the Earth BioGenome Project (EBP) standards, with a summary metric of 6.C.Q62, denoting the high quality of the data.
4. Relevance and context: The background section effectively establishes the biological significance of the *A. levana *genome. The seasonal polyphenism of this butterfly is a classic and fascinating case study, and the authors make an argument for how this genome will facilitate future research into the genetic basis of this trait.
5. Clear visualizations: Figures 2, 3, 4, 5, and 6 are highly effective. The Hi-C contact map (Figure 2) and the Merian element painting (Figure 3) provide clear visual evidence of the successful chromosome-level scaffolding and a valuable evolutionary context. The Snail and BlobToolKit plots (Figures 5 and 6) are standard and informative, effectively summarizing key assembly metrics and quality.
6. Open data and reproducibility: The article is open access, and the authors have made all raw data, assemblies, and software pipelines publicly available, which is commendable and fully aligns with FAIR data principles.

**Suggestive inputs for improvement**

 While the preprint is already of a very high standard, a few additions or clarifications could further enhance its value.

1. *Expanded abstract*: The current abstract is very concise. It would be beneficial to slightly expand it to include the key quality metrics (e.g., BUSCO score, N50, and EBP metric). This would give readers a more complete picture of the assembly's quality at a glance. For example, explicitly stating that the assembly is "chromosome-level and meets Earth BioGenome Project standards" would be a powerful summary statement.
2. *Interpretation of merian element painting*: The Merian element painting (Figure 3) is a great addition, as it shows the conservation of ancient lepidopteran linkage groups. A brief discussion of the results in the main text would be helpful. For example, are any of the identified fusions or fissions novel or consistent with other closely related Nymphalidae species? Highlighting specific observations, such as the fusion of M17 and M20 on chromosome 3, and M1 and M2 on chromosomes 1 and 2, could provide deeper insights into the chromosomal evolution of *A. levana*.
3. *Comparison with other Nymphalidae genomes*: The authors mention that *A. levana* is in the Nymphalidae family. A brief comparison of key genomic features (e.g., genome size, chromosome number) with other sequenced Nymphalidae genomes (e.g., *Heliconius*, *Danaus plexippus*) would provide valuable context. This would help position this new assembly within the broader evolutionary landscape of butterflies.
4. *Heterozygosity and haplotype assembly:* The authors assembled two haplotypes. While Haplotype 1 is well-curated, Haplotype 2 is only at the scaffold level. The reasons for this disparity could be briefly mentioned. For instance, were there technical challenges with Haplotype 2? A more detailed explanation of why one haplotype was chosen for the chromosome-level curation over the other would be a good addition. The authors could also provide more detail on how the 0.48% heterozygosity estimate from GenomeScope2.0 influenced the assembly strategy and quality.
5. *Future directions:* While the authors state that the genome will be invaluable for studying polyphenism, a concluding sentence or short paragraph outlining a few specific research questions that could be addressed with this genome would be a strong finish. For example: "This genome will be critical for identifying candidate genes involved in the photoperiodic and hormonal pathways that regulate seasonal polyphenism, as well as for comparative genomic studies across other polyphenic Nymphalidae species." This would further underscore the article's impact and value.

Overall, a good piece of work is depicted in this preprint. The suggestions are minor and primarily aimed at enriching the context and interpretation of the already robust data. This reference genome is a significant contribution to lepidopteran genomics and more specifically, the emerging area of biodiversity genomics.

Are sufficient details of methods and materials provided to allow replication by others?

Partly

Is the rationale for creating the dataset(s) clearly described?

Yes

Are the datasets clearly presented in a useable and accessible format?

Partly

Are the protocols appropriate and is the work technically sound?

Yes

Reviewer Expertise:

Biodiversity genomics, evolutionary biology and genomics.

I confirm that I have read this submission and believe that I have an appropriate level of expertise to confirm that it is of an acceptable scientific standard.

## References

[1] : The genome sequence of the Map, Araschnia levana (Linnaeus, 1758) (Lepidoptera: Nymphalidae). *Wellcome Open Research* .2025;10: 10.12688/wellcomeopenres.24633.1 10.12688/wellcomeopenres.24633.1 39944230
